# How many squat–stand manoeuvres to assess dynamic cerebral autoregulation?

**DOI:** 10.1007/s00421-018-3964-2

**Published:** 2018-08-20

**Authors:** S. C. Barnes, N. Ball, V. J. Haunton, T. G. Robinson, R. B. Panerai

**Affiliations:** 1 Department of Cardiovascular Sciences, University of Leicester, Room 210, Robert Kilpatrick Clinical Sciences Building, PO Box 65, Leicester, LE2 7LX UK; 2 National Institute for Health Research (NIHR) Leicester Biomedical Research Centre, University of Leicester, Leicester, UK

**Keywords:** Cerebral blood flow, Squat–stand manoeuvres, Transfer function analysis, Autoregulation index, Transcranial Doppler ultrasound

## Abstract

**Purpose:**

Squat–stand manoeuvres (SSMs) have been used to induce blood pressure (BP) changes for the reliable assessment of dynamic cerebral autoregulation. However, they are physically demanding and thus multiple manoeuvres can be challenging for older subjects. This study aimed to determine the minimum number of SSMs required to obtain satisfactory coherence, thus minimising the subjects’ workload.

**Method:**

20 subjects performed SSMs at a frequency of 0.05 Hz. End-tidal CO_2_, cerebral blood flow velocity, heart rate, continuous BP and the depth of the squat were measured. 11 subjects returned for a repeat visit. The time points at which subjects had performed 3, 6, 9, 12 and 15 SSMs were determined. Transfer function analysis was performed on files altered to the required length to obtain estimates of coherence and the autoregulation index (ARI).

**Results:**

After three SSMs, coherence (0.05 Hz) was 0.93 ± 0.05, and peaked at 0.95 ± 0.02 after 12 manoeuvres. ARI decreased consecutively with more manoeuvres. ARI was comparable across the two visits (*p* = 0.92), but coherence was significantly enhanced during the second visit (*p* < 0.01). The intra-subject coefficients of variation (CoV) for ARI remained comparable as the number of manoeuvres varied.

**Conclusions:**

This analysis can aid those designing SSM protocols, especially where participants are unable to tolerate a standard 5-min protocol or when a shorter protocol is needed to accommodate additional tests. We emphasise that fewer manoeuvres should only be used in exceptional circumstances, and where possible a full set of manoeuvres should be performed. Furthermore, these results need replicating at 0.10 Hz to ensure their applicability to different protocols.

## Introduction

Cerebral autoregulation (CA) is the process by which the brain is provided with relatively stable cerebral blood flow (CBF), despite fluctuations in blood pressure (BP) (Aaslid et al. 1989). It can be considered as either static (sCA) or dynamic (dCA), which refers to the response to long-term (minutes) and acute changes (seconds) in BP, respectively (Tiecks et al. 1995). Whilst there is no accepted gold standard for the quantification of dCA, transfer function analysis (TFA) is widely used (Claassen et al. 2016), alongside the autoregulation index (ARI) (Tiecks et al. 1995).

Three parameters are yielded from TFA; phase describing the temporal relationship between CBF velocity (CBFV) and BP waveforms at a given frequency, gain providing a measure of the changes in the amplitude of the CBF waveform after a change in BP, and coherence, expressing the fraction of output power that can be linearly explained by the input signal at each frequency (Zhang et al. 1998; Panerai et al. 1998; Giller 1990; Tzeng et al. 2012). When coherence is low, the reliability of phase and gain estimates reduce (Claassen et al. 2016), often leading to rejection of recordings (Zhang et al. 1998). To avoid this, coherence can be increased by inducing BP oscillations through a variety of techniques including the Valsalva manoeuvre (Hetzel et al. 2003), cyclic thigh-cuff deflation (Aaslid et al. 1989), oscillatory lower body negative pressure (Hamner et al. 2004), sit–stand manoeuvres (Sorond et al. 2008) and repeated squat–stand manoeuvres (SSMs) (Claassen et al. 2009b; Barnes et al. 2017a).

SSMs have been shown to induce large and periodic oscillations in both BP and CBFV, which has been found to greatly improve coherence, and therefore, the reliability of TFA metrics (Claassen et al. 2009b; Smirl et al. 2015; Barnes et al. 2017a). Furthermore, they have been found to produce reproducible metrics of dCA (Barnes et al. 2017a; Smirl et al. 2015). However, they are physically demanding. While younger cohorts have been able to perform SSMs, and some studies have achieved good success rates with older cohorts (Smirl et al. 2014a, 2015), some have excluded a proportion of older subjects on the grounds of musculoskeletal impairment (Zhang et al. 2009; Claassen et al. 2009a; Oudegeest-Sander et al. 2014). While it may be easier for participants to use a sit–stand manoeuvre in these instances, the levels of coherence produced are much lower than those produced by a SSM (Oudegeest-Sander et al. 2014). However, if SSMs are to be used to induce BP oscillations for the assessment of dCA in a heterogeneous and frail patient population, it would be useful to minimise the number of SSMs that are required whilst maintaining the particularly high levels of coherence achieved by these manoeuvres.

In this study, we analysed a previously collated dataset (Barnes et al. 2017a) to determine the minimum number of SSMs that are required to achieve the high levels of coherence reported in previous studies, thus minimising the exertion experienced by participants. Furthermore, we assessed the variation in ARI that occurs as different numbers of SSMs are performed to determine whether performing fewer manoeuvres affects our measures of dCA. Finally, we analysed the reproducibility of our estimates of ARI and coherence across two visits for a subset of our subjects.

## Methods

### Subjects

This analysis was based on a previously collated dataset (Barnes et al. 2017a), during which 20 subjects (mean age 22.2 ± 2.1 years, 14 male) performed SSMs. 11 subjects completed a repeat visit, but data from 1 of these subjects were not of acceptable quality. Ethical approval was obtained from the University of Leicester ethics committee (Ref: 8442-vjh12-cardiovascular sciences), and written informed consent was obtained from all participants. Detailed description of measurement techniques and protocols has been reported previously (Barnes et al. 2017a), and will, therefore, be summarised in brief.

### Instrumentation

Heart rate was measured using three-lead ECG. Nasal capnography was used to measure end-tidal CO_2_ (EtCO_2_, Salter labs, ref. 4000), and estimates of beat-to-beat BP were obtained through arterial-volume clamping of the digital artery in the right hand (Finometer, FMS, Amsterdam, Netherlands). 2 MHz Doppler probes, held in position at a fixed angle over the left and right temporal windows using a custom-designed head frame, were used to obtain estimates of CBFV in the middle cerebral arteries (MCAs). A tilt-sensor (QG-KI-090AI-K, DIS Sensors, Oostergacht, The Netherlands), fixed to the participant’s right thigh, was used to measure the angle of the squat manoeuvre. The right hand was supported with a sling to minimise movement throughout the manoeuvres to increase the reliability of the Finapres readings. Finally, brachial BP was measured intermittently using a validated sphygmomanometer (UA767 BP monitor) for calibration of the Finapres recordings.

### Squat protocol

Each subject performed 15 SSMs at a frequency of 0.05 Hz. The series of SSMs was preceded and followed by 90 s of standing quietly. Visual cues given by a computer programme were used to guide the SSMs to ensure subjects performed the manoeuvres at the correct frequency without auditory stimulation. When performing the SSMs, subjects were instructed to squat down as low as they felt able. They were informed that they would need to perform 15 squats, and to take this into account when choosing their depth. Throughout each recording, subjects were asked to breathe through their nose and to avoid a Valsalva-like manoeuvre during the SSM.

### Data analysis

Data were visually inspected; non-physiological spikes in CBFV were removed through linear interpolation. The Welch method was employed for smoothing spectral estimates using segments with 256 samples (50.12 s), with 50% superposition, to allow estimates with a minimum duration of 60 s. The time points at which subjects had performed 3, 6, 9, 12 and 15 SSMs was determined using custom-designed software, and TFA was subsequently performed on files altered to the required length. The 90 s period where subjects stood quietly at the beginning of the recording was excluded from analyses as the proportion of the file accounted for by this period notably varied as increasing numbers of SSMs were included.

The coherence function reflects the amount of output (CBFV) power that can be linearly explained by the corresponding input power (BP) at each frequency. For SSMs, coherence was analysed as point estimates at 0.05 Hz; the driven frequency of BP oscillations. As this frequency falls in the interval of 0.01–0.07 Hz, it is usually representative of the very-low frequency (VLF) band (Claassen et al. 2016). Using the inverse fast Fourier transform, the CBFV response to a step change in BP was also derived (Panerai et al. 1998). The CBFV step response was compared with ten template curves proposed by Tiecks (Tiecks et al. 1995) and the best fit curve corresponded to the ARI. The ARI after 15 SSMs was used as a reference against which the effect of reducing the number of manoeuvres was compared.

### Statistical analysis

Wilcoxon paired tests were used to compare TFA metrics from 3 vs. 6, 6 vs. 9, 9 vs. 12 and 12 vs. 15 SSMs. Repeated measures ANOVA and a Bland–Altman analysis were performed in the ten subjects who repeated the procedure at a second visit to the lab. Within-subject coefficients of variation (CoV) of the standard error of the mean were calculated to assess reproducibility. Values of *p* < 0.05 were adopted to indicate statistical significance and Bonferroni correction was applied to correct for multiple comparisons.

## Results

### Minimum SSM analysis

**Fig. 1:**
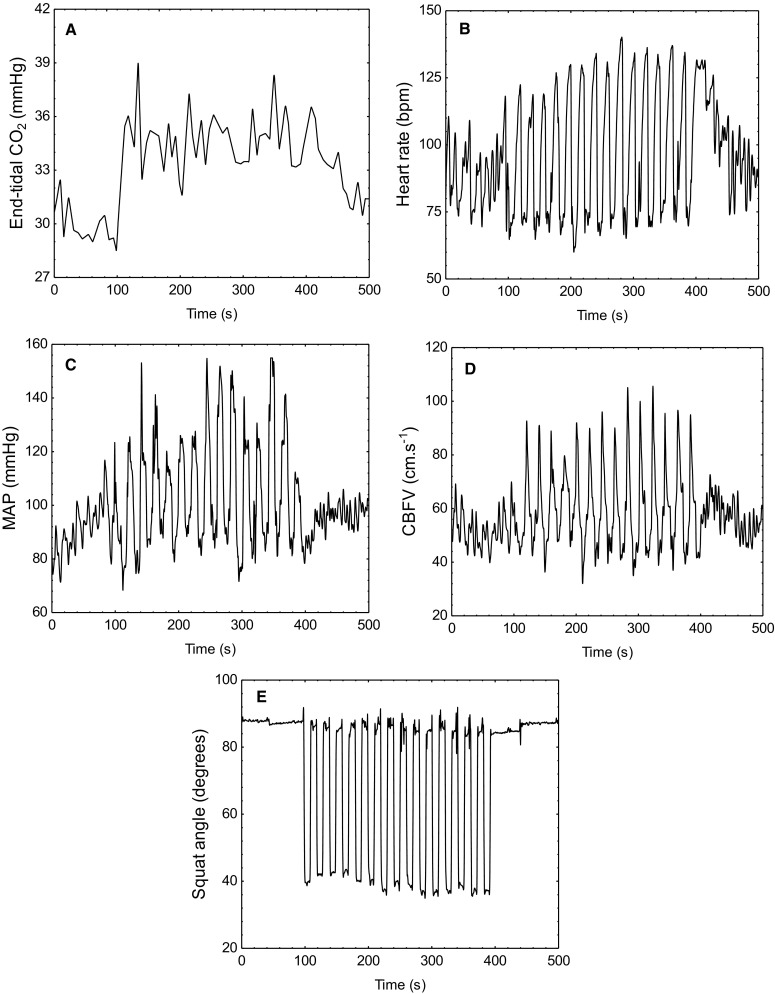
Representative changes in end-tidal CO_2_ (**a**), heart rate (**b**), mean arterial pressure (**c**), cerebral blood flow velocity (**d**) and the angle of the thigh to the horizontal (**e**) in response to a fixed-frequency squat–stand manoeuvre in a 21-year-old female subject

**Fig. 2:**
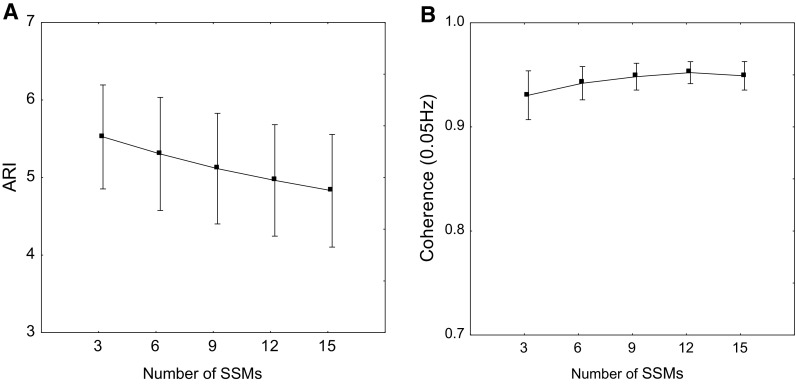
Estimates of mean ARI (**a**) and 0.05 Hz mean coherence (**b**) according to number of (0.05 Hz) SSMs. Error bars represent ± 1 SD

Representative data from one subject are shown in Fig. 1, and averaged physiological parameters for all 20 subjects are given in Table 1. Heart rate and EtCO_2_ showed highly significant trends, although of limited physiological relevance. CBFV showed a borderline difference for the right MCA (*p* = 0.08), but a significant difference for the left hemisphere. Other parameters, as detailed in Table 1, including the depth of the squat manoeuvre, did not show any differences with the increasing number of manoeuvres. Considering all subjects, coherence (0.05 Hz) was 0.93 ± 0.05 after only three SSMs. Coherence peaked at 0.95 ± 0.02 after 12 SSMs, but did not differ significantly from corresponding values after 9 or 15 SSMs. ARI was highest after three SSMs and decreased consecutively as more SSMs were performed, with significant differences for 6 vs. 9, 9 vs. 12 and 12 vs. 15 manoeuvres (*p* < 0.01 for all). Values of ARI and coherence are detailed in Table 2 and Fig. 2.

**Table 1:** Physiological parameters according to the number of SSMs performed

Parameters	Number of manoeuvres					*p* value
	3	6	9	12	15	
Right MCA CBFV (cm s^− 1^)	59.1 ± 10.3	59.9 ± 11.9	60.3 ± 13.1	60.5 ± 14.0	60.5 ± 14.6	0.08
Left MCA CBFV (cm s^− 1^)	56.3 ± 10.1	57.3 ± 11.5	57.8 ± 12.5	58.1 ± 13.4	58.3 ± 14.0	< 0.01
Mean arterial pressure (mmHg)	96.2 ± 13.1	98.7 ± 14.5	98.8 ± 15.5	98.9 ± 16.3	98.7 ± 16.7	0.22
Systolic blood pressure (mmHg)	133.6 ± 19.1	135.7 ± 20.5	136.1 ± 21.5	136.2 ± 22.2	135.9 ± 22.8	0.09
Diastolic blood pressure (mmHg)	83.9 ± 10.6	84.0 ± 11.9	83.8 ± 12.8	83.7 ± 13.3	83.4 ± 13.8	0.60
Heart rate (bpm)	91.9 ± 15.1	92.6 ± 16.5	93.9 ± 17.2	95.3 ± 17.5	96.5 ± 17.1	< 0.001
End-tidal CO_2_ (mmHg)	37.1 ± 2.3	37.9 ± 2.2	38.3 ± 2.3	38.4 ± 2.3	38.6 ± 2.3	< 0.001
Squat angle (degrees from horizontal)	24.5 ± 16.8	24.2 ± 17.3	23.2 ± 18.0	23.5 ± 17.3	25.0 ± 18.7	0.08

Values are mean ± SD

*MCA* middle cerebral artery, *CBFV* cerebral blood flow velocity, *p* value repeated measures ANOVA

**Table 2:** Variation in autoregulation index and coherence (0.05 Hz) during squat–stand manoeuvres

Parameters	Number of manoeuvres				
	3	6	9	12	15
ARI	5.52 ± 1.43	5.30 ± 1.56	5.11 ± 1.52^a^	4.96 ± 1.54^a^	4.82 ± 1.55^a^
Coherence (0.05 Hz)	0.93 ± 0.05	0.94 ± 0.03	0.95 ± 0.02	0.95 ± 0.02	0.95 ± 0.03

Values are mean ± SD

*ARI* autoregulation index

^a^Significantly different from preceding column (*p* < 0.025), as measured by Wilcoxon paired test

### Reproducibility analysis

**Fig. 3:**
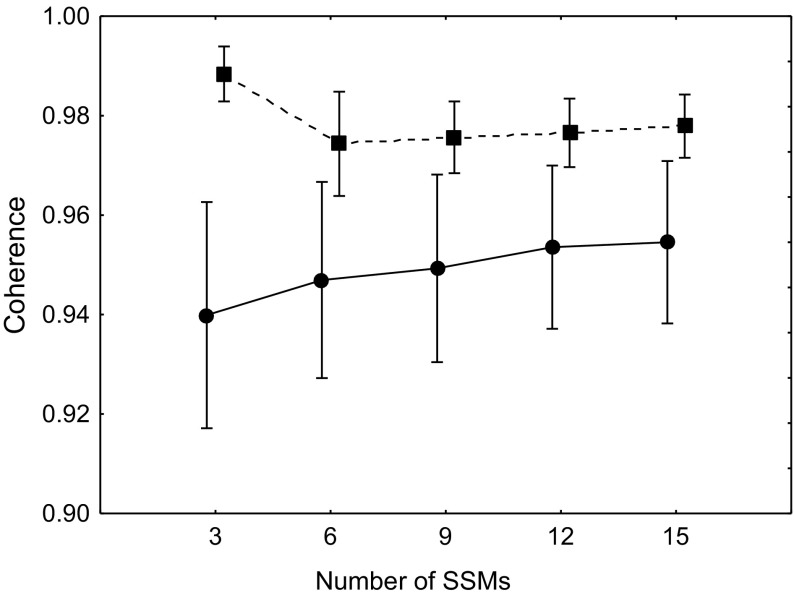
Mean 0.05 Hz coherence across two visits for ten repeat subjects. Visit 1, solid line. Visit 2, dashed line. Error bars represent ± 1 SD

**Fig. 4:**
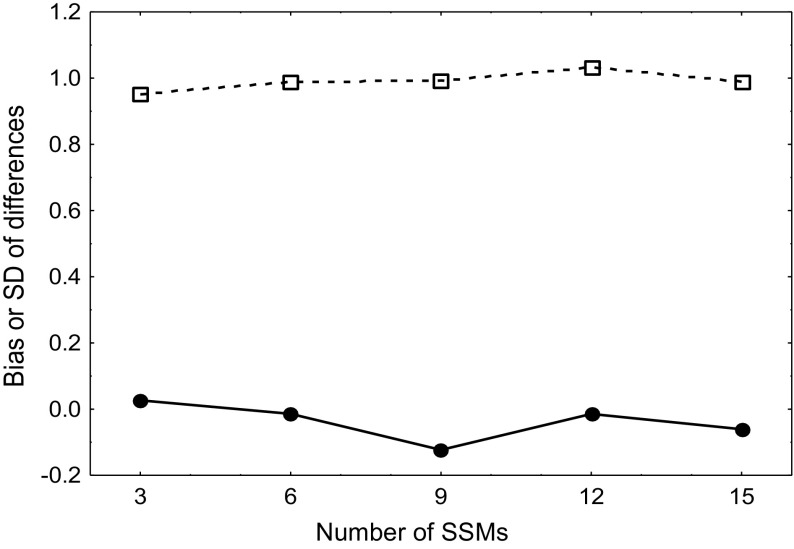
Bland–Altman plot demonstrating bias (solid line) and SD of differences (dashed line) for ARI between visits

Estimates of ARI were comparable between the two visits (*p* = 0.92). For both visits, ARI decreased consecutively as the number of manoeuvres increased (*p* = 0.003). Mean coherence was significantly increased in the second visit compared to the first (*p* < 0.01), although all values were above 0.90 for all numbers of manoeuvres across both visits (Fig. 3). Values for CoV for different numbers of manoeuvres can be found in Table 3. Neither the bias nor the limits of agreement of the Bland–Altman analysis changed as the number of manoeuvres varied (Fig. 4).

**Table 3:** Coefficients of variation (%) for ARI between visits

Number of manoeuvres	3	6	9	12	15
ARI	5.50	5.95	6.20	6.76	6.67
Coherence	1.80	1.07	1.02	0.88	0.87
Phase	6.68	5.17	5.17	4.30	3.72
Gain	23.0	20.6	20.4	19.7	19.6

*ARI* autoregulation index. Values are given as percentages

## Discussion

SSMs have been broadly well tolerated in a variety of cohorts, including heart transplant patients (Smirl et al. 2014a), subjects with Alzheimer’s disease (Claassen et al. 2009a) and healthy older adults (Smirl et al. 2015). However, some older subjects may be unable to perform these manoeuvres due to musculoskeletal difficulties, with exclusion rates varying from 16 to 50% (Oudegeest-Sander et al. 2014; Claassen et al. 2009a; Zhang et al. 2009). If SSMs are to be used regularly in patient cohorts, it would be useful to minimise the number of manoeuvres that need to be performed, to allow previously excluded patients to participate and to increase applicability.

**Fig. 5:**
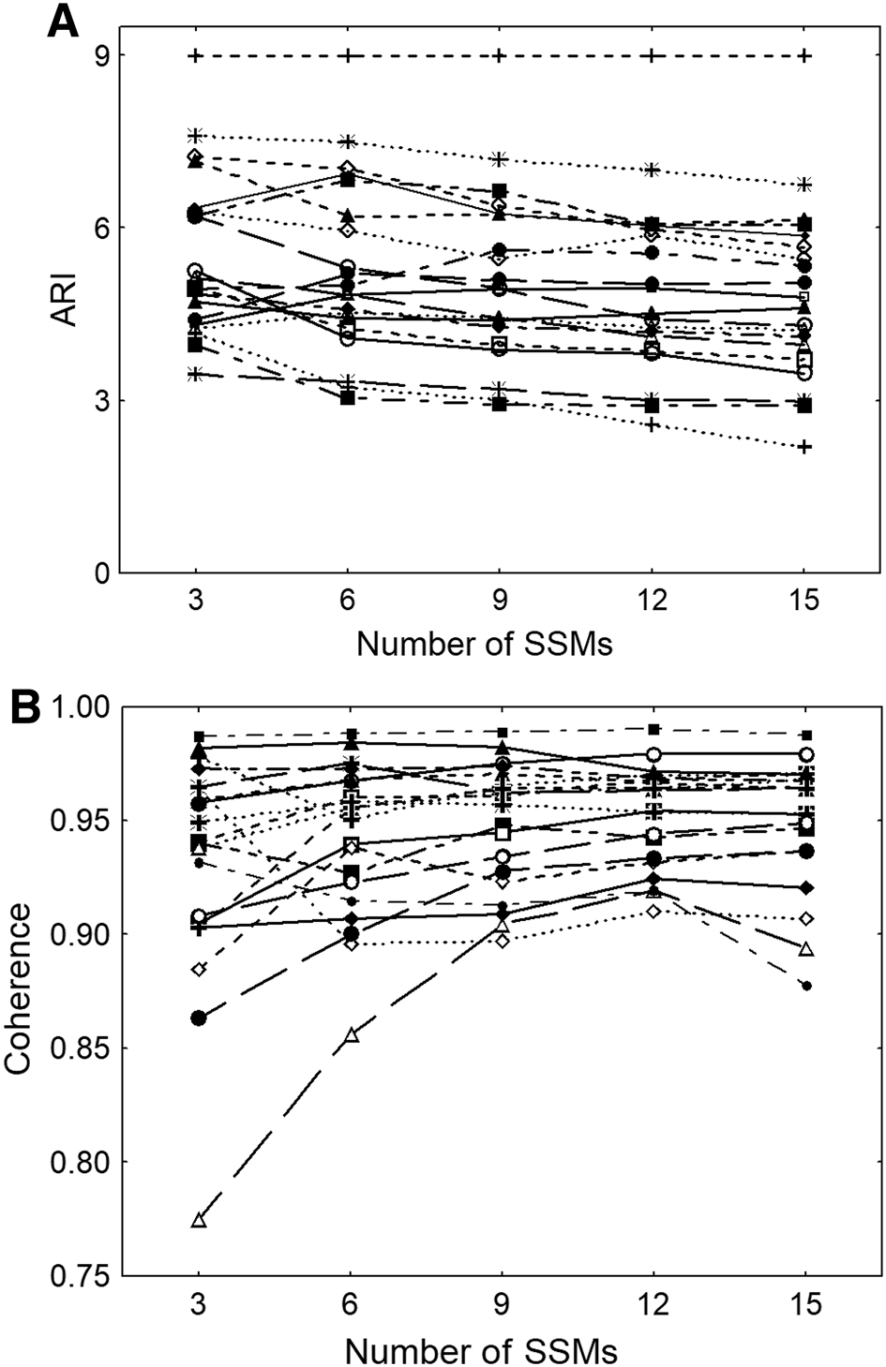
Case profiles to demonstrate the maintenance of rank for **a** ARI and **b** coherence for different numbers of SSMs

The main finding of our study was that reducing the number of SSMs led to sufficiently high values of coherence (Panerai et al. 2016; Claassen et al. 2016). After 3 SSMs, coherence (0.05 Hz) was 0.93, well above the suggested threshold of 0.50 (Claassen et al. 2016). For a small minority of our subjects, some coherence values fell below 0.90 (Fig. 5), but all values remained well above 0.50 and the majority were > 0.95. This gives investigators the option of asking frail participants to perform fewer manoeuvres than they would a physically fit subject.

Previous work analysing the repeatability of dCA metrics generated from SSMs has used the CoV or the intraclass correlation coefficient (Barnes et al. 2017a; Smirl et al. 2015). Both studies found the metrics of dCA to be broadly reproducible between visits. In the present study, we found estimates of ARI to be comparable between visits when analysed with repeated measures ANOVA, which is in keeping with the literature. Furthermore, when we performed Bland–Altman analysis, neither bias nor limits of agreement was affected by the number of manoeuvres (Fig. 4). A CoV of 20% has been suggested as the threshold for acceptable reproducibility (Smirl et al. 2015). The majority of CoV values fell well below this threshold (Table 3), adding further weight to the reproducibility of dCA metrics generated by SSMs. The exception was gain, which had CoV values marginally above and marginally below the suggested 20% threshold. However, gain has been shown previously to be a poorly reliable and poorly reproducible metric of dCA (Barnes et al. 2017a; Claassen et al. 2016), and so the values reported in the current study are comparable to that in the literature.

Interpretation of results for the ARI is more complex due to the lack of a ‘gold standard’ for dCA parameters. This limitation is not restricted to the ARI, but also applies to TFA phase and other parameters proposed for assessment of sCA and/or dCA (Claassen et al. 2016; Patel et al. 2016; Tzeng et al. 2012; Xiong et al. 2017). In the absence of a standard, we can use ARI values derived from 15 SSMs as a reference, based on current practise (Barnes et al. 2017a), to compare with values obtained from a reduced number of SSMs. Our results showed that ARI increased continuously with the reduction in the number of manoeuvres (Fig. 2a). This may be due to reduced fatigue as the subject performs fewer manoeuvres; Ogoh found that dCA was impaired during exhaustive exercise (Ogoh et al. 2005a, b). However, others have found no change in dCA with progressive workload (Brys et al. 2003). In the current study, there was no change in the squat angle achieved by participants as more manoeuvres were performed (Table 1). It is possible that the change in ARI is more representative of a central fatigue which is not reproduced in the peripheral musculature. This explains the progressive decrease in ARI despite no evidence of peripheral fatigue. Peripheral effects were also manifested by the gradual increase in heart rate and EtCO_2_ as the number of manoeuvres increased. Despite the relatively small changes in these parameters with the increased number of manoeuvres, these effects are important to be kept in mind in clinical applications where tiredness or physical limitations might have a greater physiological effect. As for the changes observed in CBFV, particularly in the left MCA, these were more likely to be due to transient changes in the first three manoeuvres, before a more stable sequence of squat–stand is established. Again, the differences showed in Table 1, although statistically significant, are of little physiological relevance.

On one hand, investigators might shy away from adopting a reduced number of SSMs that will lead to significantly different values of ARI in comparison with those obtained from 15 SSMs. On the other hand, new reference values could be established for six manoeuvres, as an example. This would facilitate the use of SSMs across a wider range of subjects, including patients that might not tolerate a 5-min protocol involving 15 SSMs. With this dilemma, of considerable relevance is the stability of relative ARI values as the number of SSMs is reduced. As shown in Fig. 5, the ranking of subjects is well maintained as the number of manoeuvres is reduced which would support the rationale for replacing the existing reference of 15 manoeuvres to 9 or fewer.

### Study limitations

First, our results only apply to SSMs at 0.05 Hz; these cannot be extrapolated to 0.10 Hz, a frequency at which many studies perform an additional set of SSMs (Smirl et al. 2014a, b, 2015; Zhang et al. 2009; Claassen et al. 2009a, b).

Second, the statistical analysis undertaken needs to be considered with caution. Classical repeated measures ANOVA to evaluate the effect of varying numbers of manoeuvres was not felt appropriate since coherence and ARI have been reported as non-Gaussian metrics in previous studies (Patel et al. 2016). Moreover, data segments for a group of sequential SSMs are included when compared with the next larger group (e.g. 3 vs. 6 and so on), so leading to a lack of independence between measurements. Consequently, we chose the Wilcoxon paired test, which ranked the parameters tested. This anticipates the needs of clinical application, for example by maintaining the ranks between controls and patients when the number of manoeuvres is changed (Fig. 5).

Third, reducing the duration of the recording used in TFA also has implications for the reliability of estimates of gain, phase and ARI. As recommended by a recent White Paper (Claassen et al. 2016), ideally, TFA should be based on 5-min duration recordings, using the Welch method to smooth spectral estimates with superposition of multiple data windows. 15 SSMs meet the requirements of generating a 15-min duration recording, but reduced numbers of SSMs will lead to a reduction in the number of data segments that can be used for spectral smoothing and for this reason could lead to biases in corresponding estimates of gain, phase and ARI. More work is needed to clarify if the gradual increase in ARI, observed with shorter SSMs durations, is a consequence of analytical bias or is a true physiological phenomenon associated with varying levels of exertion.

Finally, this is the first study to our knowledge to provide a moving estimate of ARI throughout the squat protocol. The sequential and consistent decrease in ARI as more manoeuvres were performed was unexpected, and is an important direction for future research. Future studies are needed to assess the contribution of fatigue on the estimated values of ARI with varying numbers of manoeuvres, or if these differences could be attributed to concomitant changes in cerebral or peripheral parameters that can influence dCA (Barnes et al. 2017b).

## Conclusions

The results from this analysis may help investigators in designing alternative SSM protocols, especially where participants may be unable to tolerate a standard 5-min protocol (Smirl et al. 2015; Barnes et al. 2017a), or in studies where reduced time and/or fatigue is needed to allow for additional physiological tests. Furthermore, the analysis method employed here could be used as a salvaging technique when patients fail to complete a ‘full’ series of manoeuvres. Nevertheless, it is vital that where possible, a full sequence of manoeuvres is performed to allow comparison with the standards set by previous studies. Although the CARNet guidelines (Claassen et al. 2016) for TFA of dynamic CA recommend the use of 5-min-long recordings, which would correspond to a total of 15 SSMs, it is important to note that the guidelines mainly apply to recordings of spontaneous fluctuations in BP. The results we obtained suggest that similar guidelines could be proposed for the specific case of SSMs, with the possibility of reducing the total number of manoeuvres, pending further multi-centre studies to confirm our findings.

With respect to a SSM protocol, just three SSMs were sufficient to generate values of coherence that enabled reliable estimates of gain, phase and ARI (Claassen et al. 2016; Panerai et al. 2016). We have also provided further evidence that SSMs produce reproducible estimates of dCA metrics, with the exception of gain, and this was consistent as the number of manoeuvres varied.

However, reduced manoeuvres may impact on the ability to determine the ‘best’ ARI value, and ARI estimates between subjects performing different numbers of SSMs may not be directly comparable. Whilst these results provide information to minimise the number of SSMs that need to be undertaken, and to adjust protocols accordingly, they need replicating at 0.10 Hz to ensure generalisability.

## Abbreviations

ANOVA: Analysis of variance
ARI: Autoregulation index
BP: Blood pressure
CA: Cerebral autoregulation
CBF: Cerebral blood flow
CBFV: Cerebral blood flow velocity
CoV: Coefficient of variation
dCA: Dynamic cerebral autoregulation
LF: Low frequency
SSM: Squat–stand manoeuvre
sCA: Static cerebral autoregulation
TFA: Transfer function analysis
VLF: Very low frequency
